# Do dual cognitive and motor impairments signal cognitive decline in stroke?

**DOI:** 10.1002/alz.093500

**Published:** 2025-01-09

**Authors:** Anjali Tiwari

**Affiliations:** ^1^ Colorado State Uiversity, Fort Collins, CO USA

## Abstract

**Background:**

Approximately 30% of individuals with stroke experience impending cognitive decline, often leading to dementia over the subsequent years. Despite an increased risk of dementia among individuals with stroke, there is no predictive tool to identify stroke individuals who are at higher risk of future cognitive decline. The purpose of the Come‐Drive study is to identify whether dual impairments in cognitive and motor function may provide behavioral biomarkers to predict future cognitive decline in stroke.

**Method:**

Thirty‐two stroke survivors (cognitively impaired; N = 16 and cognitively normal; N = 16) participated. All participants performed (1) Cognitive tasks: A divided attention task provided mean processing time. A simple reaction time task with the unaffected upper extremity provided an index of variability in processing time. (2) Motor tasks: Overground walking to provide gait speed and gait (stride time) variability measurements. Hierarchical logistic regression analyses were performed to determine whether processing speed and/or gait performance predicted participants’ cognitive status.

**Result:**

The cognitively impaired stroke group showed significantly increased mean processing time (p = 0.001), and increased variability in processing time (p = 0.005), compared to the cognitively normal stroke group. The cognitively impaired stroke group showed slower gait speed and amplified gait variability, but these differences failed to reach statistical significance (p > 0.05). The logistic regression showed that the mean and variability of processing time significantly predicted the cognitive status in the individuals with stroke (X^2^ (4) = 17.29, p = 0.002). Gait speed and gait variability did not emerge as significant predictors of cognitive status in individuals with stroke. The regression model accounted for 58% of the variance in determining the cognitive status post‐stroke with 83% accuracy.

**Conclusion:**

Dual decline in processing speed and gait did not emerge to be better predictors of cognitive status in individuals with stroke than the decline in processing speed alone. Perhaps a more complex motor task may serve as a better predictor of cognitive decline than a simple overground walking task. Overall, unique impairments in cognitive processing speed may serve as indicators of poor cognitive status in individuals with stroke.

